# Genetic alterations detected by comparative genomic hybridization in BRCAX breast and ovarian cancers of Brazilian population

**DOI:** 10.18632/oncotarget.25537

**Published:** 2018-06-08

**Authors:** Paula Silva Felicio, Lucas Tadeu Bidinotto, Matias Eliseo Melendez, Rebeca Silveira Grasel, Natalia Campacci, Henrique C.R. Galvão, Cristovam Scapulatempo-Neto, Rozany Mucha Dufloth, Adriane Feijó Evangelista, Edenir Inêz Palmero

**Affiliations:** ^1^ Molecular Oncology Research Center, Barretos Cancer Hospital, Barretos, SP, Brazil; ^2^ Barretos School of Health Sciences-FACISB, Barretos, SP, Brazil; ^3^ Department of Oncogenetics, Barretos Cancer Hospital, Barretos, SP, Brazil; ^4^ Department of Pathology, Barretos Cancer Hospital, Barretos, SP, Brazil

**Keywords:** BRCAX, hereditary breast cancer, hereditary breast and ovarian cancer predisposition syndrome, CNV alterations

## Abstract

**Background:**

About 5–10% of breast/ovarian cancers are hereditary. However, for a large proportion of cases (around 50%), the genetic cause remains unknown. These cases are grouped in a separated BRCAX category. The aim of this study was to identify genomic alterations in *BRCA1/BRCA2* wild-type tumor samples from women with family history strongly suggestive of hereditary breast/ovarian cancer.

**Results:**

A cohort of 31 Brazilian women was included in the study. Using the GISTIC algorithm, we identified 20 regions with genomic gains and 31 with losses. The most frequent altered regions were 1q21.2, 6p22.1 and 8p23.3 in breast tumors and Xq26 and Xp22.32-22.31 among the ovarian cancer cases. An interesting association identified was the loss of 22q13.31-13.32 and the presence of ovarian cancer cases. Among the genes present in the frequently altered regions, we found *FGFR1*, *NSMCE2*, *CTTN*, *CRLF2*, *ERBB2*, *STARD3*, *MIR3201* and several genes of *RAET* and *ULBP* family.

**Conclusions:**

In conclusion, our results suggest that alterations on chromosomes 1, 6, 8 and X are common on BRCAX tumors and that the loss on 22q can be associated with the presence of ovarian cancer.

**Methods:**

DNA copy number alterations were analyzed by 60K array comparative genomic hybridization in breast and ovarian FFPE tumors.

## INTRODUCTION

According to the World Health Organization, breast cancer (BC) is the most common tumor in women worldwide [1]. It is known that 5–10% of BC cases have a hereditary component [2], being characterized by the presence of germline mutations in the *BRCA1* [3] or *BRCA2* [4] genes, which are associated with the hereditary breast and ovarian cancer predisposition syndrome (HBOC). HBOC patients have strong personal and family histories of cancer. Moreover, these patients are characterized by early age-at-diagnosis of cancer, increased frequency of bilateral tumors, and two or more generations affected by cancer [5, 6].

Recent studies have shown that alterations in other susceptibility genes, mainly involved in the homologous recombination and DNA repair pathways, can be causal factors of hereditary breast and ovarian cancers [7]. In spite of that, the predisposing genetic cause of about 50% of the families at-risk for hereditary breast and ovarian cancers remains unknown [8, 9]. These families are grouped in a category called BRCAX.

Evidences from the literature have shown that BRCAX tumors are rather heterogeneous, involving several different histopathological subgroups and genetic alterations [10, 11]. Several authors have shown the presence of new high penetrance genes associated with breast and ovarian cancers [11–17]. However, the opinion of the scientific community is controversial. There are authors who argue that the incidence of BRCAX tumors is associated with rare syndromes in which BC is only one component [12, 15, 16]. Other authors believe that this type of tumor results from mutations in several genes with low penetrance or population-specific [11, 13, 14, 18].

Studies using array-comparative genomic hybridization (aCGH) technique suggest that several chromosomal regions are associated with the development of hereditary BC, highlighting gains at chromosomes 1q, 8q, 17q and 20q and losses within chromosomes 8p, 11q, 13q and 17p [19–24]. Despite these findings, more studies are necessary to a better understanding of BRCAX molecular events in hereditary breast cancer. In this regard, the aim of this study was to identify chromosomal and subchromosomal copy number alterations in tumor samples from Brazilian women without *BRCA1*/*BRCA2* germline mutations with family history strongly suggestive of HBOC syndrome.

## RESULTS

In the present study, we analyzed 31 Brazilian women at-risk for hereditary breast/ovarian cancer (27 with personal history of BC and 4 with ovarian tumors) without *BRCA1*/*BRCA2/TP53* germline mutations, by array-CGH. Clinicopathological characteristics and family history of the patients are specified in Table 1.

**Table 1:** Clinicopathological characteristics and family history of the patients at-risk for hereditary cancer

Family	Cancer (age at diagnosis)	Histological type	Molecular subtype	Breast/Ovarian cancer cases in the family (sex and age at diagnosis, if known)
19	Breast (44)	IDC	ER: –; PR: +; HER2: –	Sister: Breast (F,46; F,46)
29	Ovarian (42), Breast (53)	DCIS	ER: +; PR: +	Paternal side of the family: Breast (F,29); Ovarian (F,60; F,?; F, ?), Uterus (F,57; F,?; F,?); Gastric (M,42; M,?; M,?; M,?)
65	Breast (35)	IDC	ER: –; PR: –; HER2: –	Maternal side of the family: Breast (F,31; F,34; F,47; F,39; F,39; F,46)
80	Breast (43)	DCIS	ER: +; PR: +; HER2: +	Maternal side of the family: Breast (F,44; f,44; F,55; F,57; F,60), Prostate (M,?)
85	Breast (51)	IDC	ER: +; PR:+; HER2: –	Maternal side of the family: Breast (F,43; F,45; F,48), Stomach (F;45; M,56); Leukemia (M,69)
179	Breast (47)	IDC	ER: +; PR: +	Paternal side of the family: Breast (F,37; F,49; F,61), Throat (M,?; M,?)
233	Breast (49)	IDC	ER: –; PR: –; HER2: –	Maternal side of the family: Breast (F,?; F,50; F,33; F,70; F,60; F,60; F,46), Colorectal (M,65), Gastric (M,62), Pancreas (M,62), Lung (M,52; M,66; M,?)
241	Breast (45)	IDC	ER: +; PR: +; HER2: –	Paternal side of the family: Breast (F,48; F,49)
275	Ovarian (60)	Serous adenocarcinoma	Not applicable	Paternal side of the family: Breast (F,32; F,35), Prostate (M,80)
289	Breast (48)	IDC	ER: –; PR: –; HER2: +	Maternal side of the family: Breast (F,50; F,65; F,65)
306	Melanoma (26), Breast (36)	IDC	ER: +; PR: +; HER2: –	Maternal side of the family: Breast (F,43; F,?)
320	Ovarian (53)	Serous adenocarcinoma	Not applicable	Maternal side of the family: Breast (F,52), Ovarian (F,71), Uterus (F,60), Thyroid (M,29), Lung (M,83)
426	Breast (38)	IDC	ER: +; PR: +; HER2: –	Paternal side of the family: Breast (F,20); Ovarian (F,28), Leukemia (M,78), Esophagus (M,?)
494	Breast (33)	IDC	ER: +; PR: +; HER2: +	Maternal side of the family: Breast (F,38; F,?), Ovarian (F,38)
558	Breast (37)	IDC	ER: –; PR: –; HER2: –	Maternal side of the family: Breast (F,52; F,?; F,?; F,?, F,?), Ovarian (F,42), Skin (F,?)
563	Breast (39)	IDC	ER: +; PR: –; HER2: –	Paternal side of the family: Breast (F,30; F,40; F,45, F,50; F,51), Lung, (M,?), Colorectal (F,64), Skin (M,72)
581	Breast (45)	DCIS	Not available	Paternal side of the family: Breast (F,49; F,46; F,54), Prostate (M,60; M,70), head and neck (M,83)
593	Breast (39)	IDC	ER: +; PR: +; HER2: –	Paternal side of the family: Breast (F,50; F,?; F,?; F,?), Ovarian (F,?; F,?), Gastric (M,?; M,?), Colorectal (M,?; M,?)
626	Breast (46)	DCIS	ER: +; PR: +	Maternal side of the family: Breast (F,74; F,80; F,57; F,45), Ovarian (F,45), Thyroid (F,40), Skin (M,80), Pancreas (M,?), Myeloma (M,60), Lips (M,?)
638	Breast (42)	ILC	ER: +; PR: +	Maternal side of the family: Breast (F,49; F,50; F,?), Gastric (F,55), Thyroid (F,36), Lips (F,55)
649	Breast (38)	IDC	ER: +; PR: +; HER2: –	Maternal side of the family: Breast (F,64), Ovarian (F,61), Thyroid (F,61)
695	Ovarian (21)	Serous adenocarcinoma	Not applicable	Paternal side of the family: Breast (F,42), Ovarian (F,68), Colorectal (F,40; M,40), Gastric (F,50; F,70)
960	Bilateral Breast (59,70)	IDC	ER: +; PR: +; HER2: –	Maternal side of the family: Breast (F,34; F,59), Uterus (F,45), Lung (M,77; M,?)
974	Breast (46)	IDC	ER: +; PR: +; HER2: –	Maternal side of the family: Breast (F,55; F,45; F,60; F,60; F,55; F,45; F,60), Prostate (M,70; M,80)
981	Breast (37)	IDC	ER: +; PR: +; HER2: –	Maternal side of the family: Breast (F,32; F,70; F,60), Melanoma (F,30; F36), Leukemia (F,5), Bile ducts (M,49; F,55)
1014	Breast (42)	DCIS	ER: +; PR: +	Maternal side of the family: Breast (F,53; F,?); Melanoma (F,75), Lymphoma (M,19), Liver (F,?), Brain (F,?)
1024	Breast (48)	IDC	ER: –; PR: –; HER2: –	Paternal side of the family: Breast (F,70; F,72; F,44; F;44; F,49), Ovarian (F,56), Colorectal (M,20), Melanoma (M, ?), Prostate (M,50), Gastric (F,70; F,72; F,41)
1055	Ovarian (57)	Serous adenocarcinoma	Not applicable	Maternal side of the family: Breast (F,49; F,50), Pancreas (F,50), Lung (M,?)
1095	Breast (43)	IDC	ER: –; PR: –; HER2: +	Paternal side of the family: Breast (F,27; F,42), Uterus (F,98), Throat (M,72)
1151	Breast (38)	IDC	ER: +; PR: +; HER2: –	Maternal side of the family: Breast (F,35; F,60)
1264	Breast (27)	IDC	ER: –; PR: +; HER2: –	Maternal side of the family: Breast (F,50), Pancreas (M,75); Intestine (M,81)

Briefly, the mean age at BC diagnosis was 42.9 years (SD = 7.9), ranging from 27–70 years. The majority of BC was invasive ductal carcinoma (77.8%), estrogen and progesterone positive (69.2% and 73.1%, respectively) and HER2 negative (64.0%). Regarding molecular classification, the majority of patients presented luminal type tumors (21 patients, 80.8%), four patients (15.4%) had triple negative tumors and only one patient (3.8%) was diagnosed with a HER2 subtype tumor.

All four ovarian cancer patients developed serous adenocarcinoma subtype tumors. The average age at diagnosis was 47.7 years (SD = 18.0), ranging from 21–60 years.

A detailed cancer family history can be found in Table 1. All patients reported at least one case of BC in the family, diagnosed at early age (<55 years for breast cancer cases). In addition, two women at-risk for hereditary BC (samples: 960 and 1024) had a family history with bilateral BC. Among patients diagnosed with BC, the majority reported more than three cases of BC in the family history (16 cases, 59.3%). Meanwhile, all patients diagnosed with ovarian cancer, reported three or less BC cases in their families (*p* = 0.043). Moreover, 12 patients reported the presence of ovarian cancer in the family history.

**Figure 1:**
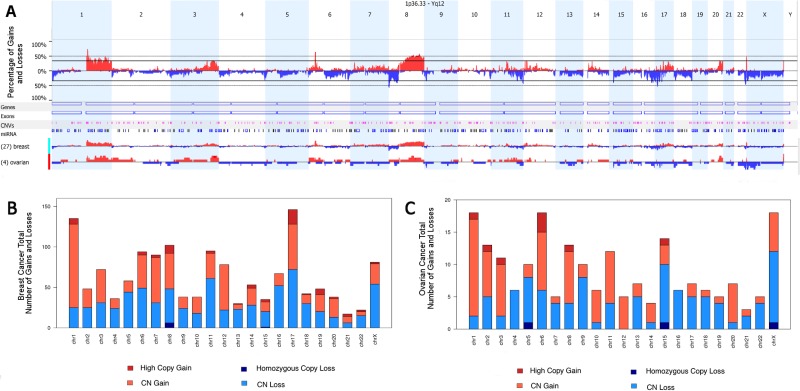
Overview of gained and lost regions across all chromosomes (**A**) Overall and specific breast and ovarian copy number aberration frequencies. Regions presenting copy gains are shown in red and with copy loss in blue. (**B**) Overview of gained and lost regions across all chromosomes in breast tumors. (**C**) Overview of gained and lost regions across all chromosomes in ovarian tumors.

The molecular analysis revealed gained and lost regions across all chromosomes for both breast and ovarian tumors (Figure 1). We found 20 gained regions and 31 lost in BRCAX tumors. In addition, some variations, although not statistically significant, were found only in patients diagnosed with breast cancer, such as: gains of 7p22.1, 12p13.1, 14q13.3-q21.1, 17q11.2, 17q12 and 17q21.32-q21.33, and losses of 2p25.3, 6q25.3-q26 and 10q26.3. Moreover, the gain of Xq26 and loss of Xp22.32–22.31 were more frequent in ovarian cancer (100%), compared with breast cases (26% and 59%, respectively) (*p* = 0.01 for both regions). Loss of 22q13.31–13.32 was detected more often in ovarian than in breast cancer cases (*p* = 0.043). In addition, a significant number of copy number alterations involving chromosome 8 was observed.

When family history was taken into consideration for copy number variation analyses, we observed that loss of 22q13.31–13.32 region was significantly associated with the presence of ovarian family history (*p* = 0.03). This region includes *MIR3201*, *LOC284933*, *FAM19A5*, *MIR4535*, *LINC01310* genes. Other significant association found included gains in the 6p22.1 region (including 13 histone family genes) in 100% of metastatic cases (*p* = 0.03). Finally, we found loss of 6q25.1 in 71% of patients with metastasis (*p* = 0.01). This region includes *RAET1E*, *RAET1E-AS1*, *RAET1G*, *ULBP2*, *ULBP1*, *RAET1K*, *RAET1L*, *ULBP3*, *PPP1R14C*, *IYD*, *PLEKHG1*, *MTHFD1L* genes (Supplementary Table 1).

**Figure 2:**
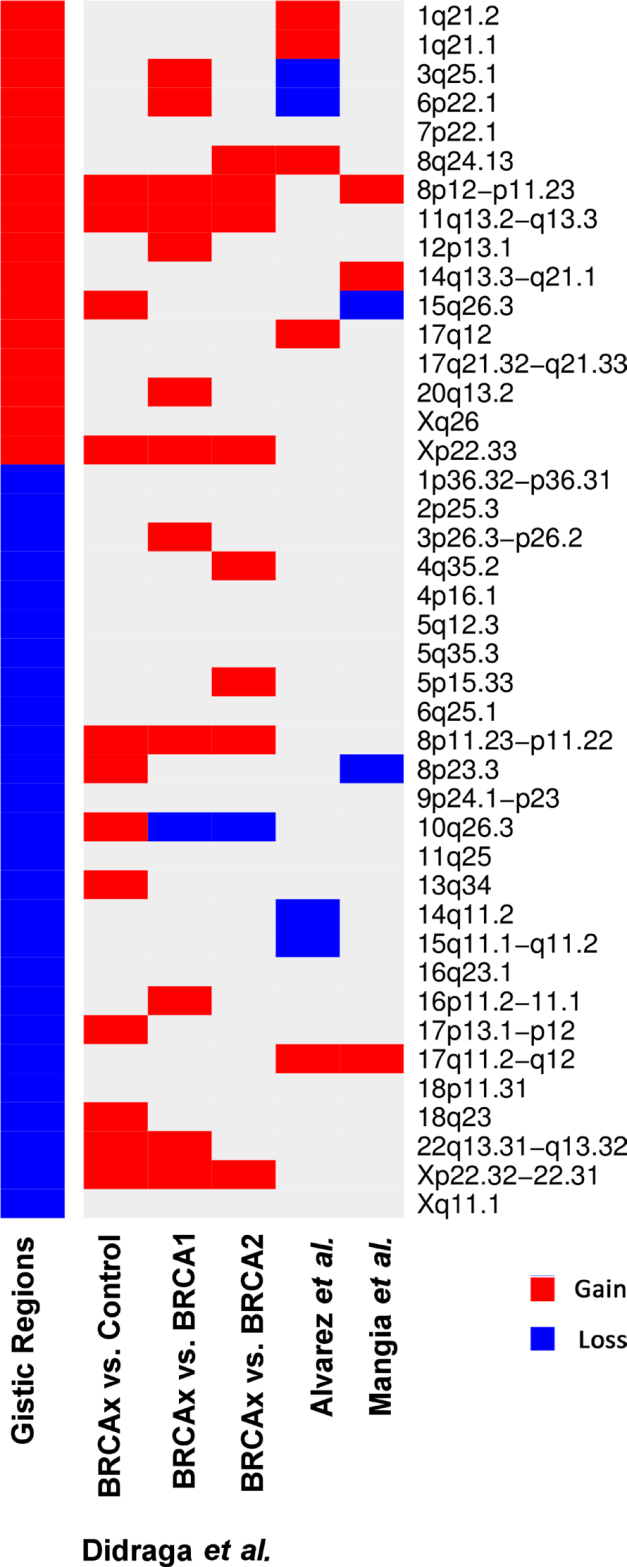
Heatmap representing the gains (in red) and losses (in blue) through aCGH found by GISTIC algorighm in common with previous studies by Didraga *et al.* (2011), Alvarez *et al.* (2016) and Mangia *et al.* (2008)

In addition, when comparing our findings with those of the literature of BRCAX tumors, we observed that our results corroborate some findings reported by Didraga *et al.* (2011), Alvarez *et al.* (2016) and Mangia *et al.* (2008), showing 50%, 21% and 12% of common regions, respectively (Figure 2).

Finally, we found that 22 genes present in gained regions also present overexpression in the Oncomine database, whereas 21 genes present in lost regions show loss of expression in the same database (*p* < 0.01, Table 2).

**Table 2:** Genes in gained regions that presented *in silico* overexpression and genes in lost regions that presented *in silico* loss of expression

Event^1^	Cytoband	Genes
Gain/Overexp	1q21.1-q21.2	*PEX11B, PDE4DIP, ECM1, TARS2, RPRD2*
Gain/Overexp	6p22.1	*HIST1H3H, HIST1H4J, HIST1H4K*
Gain/Overexp	7p21.1	*HDAC9*
Gain/Overexp	8p11.23-p11.22	*TM2D2, LETM2, RNF5P1, FGFR1*
Gain/Overexp	8q24.13	*NSMCE2, KIAA0196*
Gain/Overexp	11q13.3	*CTTN*
Gain/Overexp	17q12	*ERBB2, STARD3, GRB7*
Gain/Overexp	17q21.32-q21.33	*PHB, ABI3*
Gain/Overexp	Xp22.33	*CRLF2*
Loss/LOExp	1p36.32	*TPRG1L, AJAP1*
Loss/LOExp	2p25.3	*FAM150B, TMEM18, TPO*
Loss/LOExp	3p26.3-p26.2	*CRBN, CNTN4*
Loss/LOExp	5q35.3	*ADAMTS2, ZNF879, COL23A1*
Loss/LOExp	8p23.3	*ERICH1, RPL23AP53, OR4F21, ZNF596*
Loss/LOExp	11q25	*JAM3, LOC283177, THYN1*
Loss/LOExp	14q11.2	*OR4K5*
Loss/LOExp	16q23.1	*CNTNAP4, SYCE1L*
Loss/LOExp	Xp22.32-p22.31	*NLGN4X*

^1^Events represent Gain or Loss in our samples with concurrent Overexpression (Overexp) or Loss of Expression (LOExp) on Oncomine samples.

## DISCUSSION

In the present study, a BRCAX tumor characterization of FFPE samples has been performed by array comparative genomic hybridization. Among the altered loci, we can highlight the identification of several alterations in chromosome 8, including losses on 8p12-p11.23 and gains on 8p12-p11.23 and 8q24.13, in concordance with previous studies of BRCAX tumors [14, 25, 26]. Besides, the chromosomal region 8p12-p11 has been reported to be amplified in 10–23% of BC cases [27–29], and some studies have shown that amplification on this region is associated with poor clinical outcome [27, 30]. We found by *in silico* analysis that 4 genes present in this region (including *FGFR1* and *NSMCE2)* are overexpressed.

The *FGFR1* gene encodes a transmembrane protein that interacts with fibroblast growth factors and directly influence mitogenesis and cell differentiation. In fact, there are several studies showing different treatment outcomes of breast cancer women depending on the *FGFR1* status [31–33]. Similarly, *NSMCE2* plays an important role in cell cycle, since its depletion in MCF-7 breast cancer cells affected cell cycle and G1-S transition [34]. Moreover, the overexpression of cortactin (*CTTN*), present in 11q13.3, was linked to *CCND1* amplification in premenopausal breast cancer [35], although it failed to demonstrate a strong prognostic value in patients with breast cancer [36]. Conversely, its upregulation promoted colon cancer progression through ERK pathway [37]. Therefore, other studies have shown that amplification on chromosomal region 8p12-p11 in combination with amplification on 11q13 have more impact on patient outcome than amplification on only one of the two loci [27, 38].

In addition to gains and losses on chromosome 8 and alterations on chromosome 11, alterations in chromosome X seem to be characteristic of BRCAX tumors. In our study, a great number of samples showed gains on regions 11q13.2-q13.3 and Xp22.33, which were also identified by Didraga and collaborators [25]. Although it is not extensively studied in breast cancer, the overexpression of *CRLF2*, present in Xp22.33, has been demonstrated to be a marker of poor outcome of pediatric and adult B-precursor acute lymphoblastic leucemia (ALL) (as reviewed in [39]).

Study performed by Gronwald *et al.* [19] compared BRCAX with sporadic breast cancers and identified several altered regions (114 gains and 36 losses) in 18 patients. Their findings showed concordances with our results, presenting more often gains in 1q, 6p, 17q and frequent loss of 8p. Beside the well known effects of *ERBB2* amplification in breast cancer development, the overexpression of *STARD3* (located in the same locus) seems to be important, since it may contribute to increased proliferation, migration and invasion of breast cancer cells (as reviewed in [40]). Finally, considering our findings of altered regions found in BRCAX associated with metastasis (gain of 6p22.1 and loss of 6q25.1), gain of 6p was previously associated with BRCAX, and loss of 6q with *BRCA1* tumors [41]. In fact, there are several members of *RAET* and *ULBP* family present in this locus. These members are ligands of C-type lectin-like receptor NKG2D, present in NK and T cells subsets, highly involved in tumor immunosurveillance [42]. Therefore, the loss of this region may have led to lower expression of these ligands, leading to less immunogenicity of the tumor cells. In fact, there are reports on colorectal cancer that have demonstrated this same pattern, and several authors discuss the potential therapeutic utility of NKG2D ligands in the treatment of this disease [42–44]. Therefore, these alterations on chromosome 6 seem to be highly associated with breast cancer tumors and may be of interest for further studies.

We also found that loss of 22q13.31–13.32 was significantly associated with presence of ovarian tumors (in the proband or in the family). The loss of heterozygosis (LOH) of chromosome 22q has been reported in a variety of cancers, including ovarian cancers, where the LOH rates reached 70% of cases [45, 46]. Study published by Zweemer *el al.* (2001) reported a significant loss of 22q, identified through aCGH in familial ovarian tumors [47]. Interestingly, *MIR3201* was significantly downregulated in recurrent epithelial ovarian cancer (EOC), when compared to primary EOC [48]. To the best of our knowledge, there are no studies pointing to the functional relevance of *MIR3201* in ovarian cancer, however, further studies may be performed to evaluate its possible role as a biomarker of EOC recurrence.

In summary, our findings support previous data of BRCAX related alterations and point to new regions potentially associated with personal and family history of ovarian cancer. In the present study, we could identify by aCGH analysis a potentially BRCAX-associated ovarian region on chromosome 22. Given our limited sample size, further work should be performed in order to validate our findings, to identify the driver genes associated with the BRCAX tumor development, as well as to uncover the role of those altered regions in cancer formation and progression.

## MATERIALS AND METHODS

### Ethics statement

All participants gave their consent to use tumor samples for academic genetic research. In addition, the ethics committee of the Barretos Cancer Hospital (BCH) approved this study (approval number: 916/2015).

### Patients

This study included 31 unrelated Brazilian women at-risk for hereditary breast and ovarian cancer from the Oncogenetics Department of BCH. Those women were referred from the Oncogenetics Department of BCH for *BRCA1, BRCA2* and *TP53* genetic testing due to the presence of clinical criteria for HBOC, but no genetic alterations in these genes were found. For the purpose of the present study, were included only families fulfilling the following criteria: patients diagnosed with breast/ovarian cancer at an early age (<55 years), with at least two relatives with breast and/or ovarian cancer, two or more generations affected by cancer and absence of male BC.

Clinical information was obtained through detailed review of the patient´s clinical chart. For family history data, all pedigrees were revised.

### Sequencing of *BRCA1, BRCA2* and *TP53*

Analysis of the presence of germline mutations in *BRCA1*/*BRCA2/TP53* genes was conducted at the Center of Molecular Diagnosis of BCH as part of routine care through NGS sequencing followed by rearrangement analysis through MLPA (Multiplex Ligation-dependent Probe Amplification Analysis), as described elsewhere by Fernandes *et al*. [49].

### Tumor samples

For aCGH analysis, a representative section of FFPE tumor tissue from the breast or the ovarian tumor was stained by hematoxylin and eosin (H&E) and evaluated by a pathologist to verify tumor content (>70% tumor) and further microdissection.

### DNA isolation and quality control

Following microdissection, DNA extraction steps were carried out using DNeasy Blood and Tissue kit (*Qiagen*), following the manufacturer's instructions. The quality and integrity of the extracted DNA was assessed by multiplex PCR reaction using four primer pairs for the *GAPDH* gene (amplifying 100, 200, 300 and 400 bp, respectively), as described by Van Beers *et al.* [50]. The PCR reaction carried out contained (in a final volume of 30 μL) 1.5 mM MgCl_2_; 0.2 mM dNTP (*Invitrogen*); 0.133 μM of each primer; 1 U Taq DNA polymerase (*Invitrogen*) and 60 ng of tumor DNA. Reactions were performed in a *Veriti* thermocycler (*Thermo Fisher Scientific*) using the following amplification parameters: 94°C for 1 minute, 35 cycles of 94°C for 1 minute, 56°C for 1 minute, and 72°C for 3 minutes. Finally, a final extension at 72°C for 7 minutes. Amplification of DNA was verified by agarose gel electrophoresis.

### Array comparative genomic hybridization

aCGH was performed on oligonucleotide-based SurePrint G3 Unrestricted CGH 8 × 60 K microarray slides, according the protocol provided by the manufacturer. In brief, 1 μg in final volume of 13 μL of normal female control DNA – reference DNA (DNA universal control-Promega Madison WI USA- Woman Reference: G152A) and patient's DNA were differentially labeled with Cy3 (cyanine 3-deoxyuridine triphosphate) and Cy5 (cyanine 5-deoxyuridina triphosphate), respectively, using Agilent SureTag Complete DNA Labeling Kit (*Agilent Technologies*). Labeled DNA was then cleaned with purification columns (*Agilent Technologies*) and hybridized on array at 65°C for 24 hours, according to manufacturer's recommendations. Microarrays were washed using Agilent Oligo aCGH Wash Buffers and scanning was performed using Agilent SureScan Microarray Scanner according to manufacturer's instructions (*Agilent Technologies*).

### Data analysis

Data quantification of aCGH was performed with Feature Extraction software (*Agilent Technologies*) and the txt output files were imported into Nexus Copy Number v8.0 (*BioDiscovery Inc*) for visualization and downstream analysis. BioDiscovery's FASST2 Segmentation Algorithm, a Hidden Markov Model (HMM) based approach, was used to make copy number calls. The FASST2 algorithm, unlike other common HMM methods for copy number estimation, does not aim to estimate the copy number state at each probe but uses many states to cover more possibilities, such as mosaic events. These state values are then used to make calls based on a log-ratio threshold. The significance threshold for segmentation was set at 5.0E-6 also requiring a minimum of 3 probes per segment and a maximum probe spacing of 1,000 kb between adjacent probes before breaking a segment. The log ratio thresholds for single copy gain (or amplification) and single copy loss (or deletion) were set at 0.2 and -0.23, respectively. The log ratio thresholds for two or more copy gain (or high copy gain) and homozygous loss (or high copy loss) were set at 1.14 and -1.1 respectively. A 3:1 sex chromosome gain threshold was set to 1.2 and a 4:1 sex chromosome gain threshold was set to 1.7. Male sex chromosome big loss threshold was set to -1.1. GISTIC (Genomic Identification of Significant Targets in Cancer) algorithm was used within Nexus 8.0 to identify regions that are significantly amplified or deleted across a set of samples. It was considered the default parameters of Q-bound ≤ 0.05 with False Discovery Rate (FDR) correction and G-score cut-off ≤ 1.0. The identification of genes and CNVs were also performed within Nexus 8.0, being CNVs filtered according to 1000 genomes project. It was calculated the frequency of the gained and lost remaining CNVs and further separated according to >1% (rare CNVs) and ≥1% (common CNVs). The peaks identified by GISTIC algorithm were associated to breast and ovarian cancer family history and clinical characteristics, i.e. clinical staging, age at diagnosis (≤30, 31–45 and ≥45 years), molecular subtype, histological subtype, presence of metastasis and recurrence. These analyses were done by Fisher's exact test (within SPSS v.21.0 software for Windows (Chicago, IL) considering the significance level of 5%.

Besides, the genomic regions found to be significant in GISTIC were considered for further analysis using the professional version of the compendium of cancer transcriptome profiles, Oncomine™ (Compendia Bioscience, Ann Arbor, MI). There were selected 13 breast and 5 ovarian cancer datasets (totalizing over 4000 samples). For each cancer type (breast or ovary), we selected the genes that presented gain or loss in our aCGH, and considered relevant those that presented gain in our aCGH and overexpression in Oncomine (*P* < 0.01), or those that presented loss in our aCGH and loss of expression in Oncomine (*P* < 0.01).

## SUPPLEMENTARY MATERIALS TABLE





## Abbreviations

aCGH: Array comparative genomic hybridization
BC: breast cancer
BCH: Barretos Cancer Hospital
ER: estrogen receptor
HBOC: Hereditary Breast and Ovarian Cancer Predisposition Syndrome
HER2: Human Epidermal growth factor Receptor 2
PR: progesterone receptor
