# Supplementary material for: Genetic alterations detected by comparative genomic hybridization in BRCAX breast and ovarian cancers of Brazilian population
Source: Oncotarget. 2018 Jun 8;9(44):27525–34. doi: 10.18632/oncotarget.25537 (PMC6007956; doi:10.18632/oncotarget.25537)
Supplement: Supplementary file 1 [file oncotarget-09-27525-s001.pdf]

Genetic alterations detected by comparative genomic hybridization in BRCAX breast and ovarian cancers of Brazilian population

SUPPLEMENTARY MATERIALS

Supplementary Table 1: Complete list of candidate genes and CNVs by GISTIC. See Supplementary_Table_1
