# Supplementary material for: Genetic alterations detected by comparative genomic hybridization in BRCAX breast and ovarian cancers of Brazilian population
Source: Oncotarget. 2018 Jun 8;9(44):27525–34. doi: 10.18632/oncotarget.25537 (PMC6007956; doi:10.18632/oncotarget.25537)
Supplement: Supplementary file 2 [file oncotarget-09-27525-s002.doc]

**Supplementary Table 1: Complete list of candidate genes and CNVs by GISTIC**

| **Gistic regions** | **Event** | **Number of samples** | **Genes** | **CNVs < 1%** | **CNVs > 1%** |
| --- | --- | --- | --- | --- | --- |
| chr1:148,555,037-148,930,370 | Gain | 21 | PRPF3,RPRD2,MIR6878,TARS2,ECM1,FALEC,MIR4257,ADAMTSL4,ADAMTSL4-AS1,MCL1,ENSA,GOLPH3L | - | - |
| chr1:143,639,135-144,278,309 | Gain | 16 | NBPF20,PDE4DIP,NBPF12,SEC22B,NOTCH2NL,NBPF25P,LOC101928979,HFE2,TXNIP,POLR3GL,ANKRD34A,LIX1L,RBM8A,GNRHR2,PEX11B,ITGA10,ANKRD35,NBPF10 | esv3380599,esv3414198,esv3360116,esv3368223,esv3352814,esv3387252,esv3361392,esv3349227,dgv191e59 | dgv199e59,dgv200e59,esv3355910,esv3309997,esv3304560,esv3405247,esv3413349,dgv201e59 |
| chr3:151,524,863-152,116,104 | Gain | 11 | TSC22D2,SERP1,EIF2A,SELT,ERICH6,ERICH6-AS1,LOC101928105,SIAH2 | esv3342752 | - |
| chr6:27,864,081-27,957,537 | Gain | 20 | HIST1H2BL,HIST1H2AI,HIST1H3H,HIST1H2AJ,HIST1H2BM,HIST1H4J,HIST1H4K,HIST1H2AK,HIST1H2BN,HIST1H2AL,HIST1H1B,HIST1H3I,HIST1H4L | - | - |
| chr7:17,587,293-18,535,820 | Gain | 9 | SNX13,PRPS1L1,MIR1302-6,HDAC9 | esv3394688,esv3362182,esv3306132,esv3380997 | dgv3749e59,esv3374815,esv3306306,esv3354676,esv3389040,esv3306932,esv3308523,esv3304893,esv3427043,esv3441757,esv3437954 |
| chr8:126,015,907-126,691,570 | Gain | 18 | LINC00964,ZNF572,SQLE,KIAA0196,NSMCE2,TRIB1 | - | esv3342637,esv3445339 |
| chr8:38,233,025-39,318,619 | Gain | 5 | DDHD2,PLPP5,WHSC1L1,LETM2,FGFR1,C8orf86,RNF5P1,TACC1,PLEKHA2,HTRA4,TM2D2,ADAM9,ADAM32,ADAM5 | esv3433731,esv3449093 | dgv4129e59,esv3359463 |
| chr11:69,849,987-70,077,870 | Gain | 9 | PPFIA1,CTTN,SHANK2 | - | esv3381709 |
| chr12:12,630,063-12,788,197 | Gain | 11 | CREBL2,GPR19,CDKN1B,APOLD1 | - | - |
| chr14:36,796,282-37,159,041 | Gain | 10 | MIPOL1,FOXA1,TTC6 | - | esv3398904 |
| chr15:96,667,275-97,452,797 | Gain | 2 | FAM169B,IRAIN,MIR4714,IGF1R,PGPEP1L,LUNAR1 | esv3435433,esv3404889,esv3361098 | esv3408735 |
| chr17:35,060,044-35,156,109 | Gain | 10 | STARD3,TCAP,PNMT,PGAP3,ERBB2,MIR4728,MIEN1,GRB7 | - | - |
| chr17:44,648,530-44,976,930 | Gain | 6 | ABI3,PHOSPHO1,FLJ40194,MIR6129,ZNF652,LOC102724596,PHB,LOC101927207,NGFR,MIR6165,LOC100288866 | - | esv3326018, esv3307703 |
| chr20:50,878,094-51,142,697 | Gain | 10 | TSHZ2 | - | - |
| chrX:154,638,096-154,913,754 | Gain | 11 | SPRY3,VAMP7,IL9R,DDX11L16 | - | - |
| chrX:0-1,514,088 | Gain | 15 | PLCXD1,GTPBP6,PPP2R3B,SHOX,CRLF2,CSF2RA,MIR3690,IL3RA,SLC25A6,LINC00106,ASMTL-AS1,ASMTL | dgv4550e59,dgv4551e59,dgv4552e59,dgv4553e59,dgv4554e59,dgv4555e59,dgv4556e59,dgv4557e59,dgv4558e59,dgv4559e59,dgv4560e59,dgv4561e59,dgv4562e59 | esv3334914,esv3414524,esv3450882,esv3382952,esv3442681,esv3426006,esv3327828,esv3447355,esv3447400,esv3338982,esv3439707,esv3310495,esv3336590,esv3389029,esv3416240,esv3334895,esv3362005,esv3405532,esv3448400,esv3326708 |
| chr1:2,511,532-6,068,705 | Loss | 11 | FAM213B,MMEL1,TTC34,ACTRT2,LINC00982,MIR4251,PRDM16,ARHGEF16,MEGF6,MIR551A,TPRG1L,WRAP73,TP73,TP73-AS1,CCDC27,SMIM1,LRRC47,CEP104,DFFB,C1orf174,LINC01134,LINC01346,LOC284661,AJAP1,MIR4417,MIR4689,NPHP4,KCNAB2 | - | - |
| chr2:0-3,901,873 | Loss | 7 | FAM110C,SH3YL1,ACP1,FAM150B,TMEM18,LINC01115,LOC101060385,SNTG2,TPO,PXDN,MYT1L,MYT1L-AS1,LINC01250,TSSC1,TRAPPC12,ADI1,RNASEH1,RNASEH1-AS1,RPS7,COLEC11,ALLC,DCDC2C | - | - |
| chr3:2,363,239-4,305,022 | Loss | 8 | CNTN4,CNTN4-AS1,IL5RA,TRNT1,CRBN,LRRN1 | - | - |
| chr4:190,034,368-191,273,063 | Loss | 10 | LINC01262,LINC01596,LOC283788,FRG1,FRG1CP,FRG2,DBET | - | - |
| chr4:7,135,530-7,702,329 | Loss | 7 | FLJ36777,MIR4798,PSAPL1,MIR4274,SORCS2 | - | - |
| chr5:65,956,884-66,166,332 | Loss | 7 | MAST4 | - | - |
| chr5:177,726,909-178,886,217 | Loss | 7 | COL23A1,CLK4,ZNF354A,AACSP1,ZNF354B,ZFP2,ZNF454,GRM6,ZNF879,ZNF354C,ADAMTS2 | - | - |
| chr5:2,075,794-3,822,399 | Loss | 8 | LOC100506858,IRX2,C5orf38,LINC01377,LINC01019,LINC01017,IRX1 | - | - |
| chr6:150,238,707-151,397,838 | Loss | 9 | RAET1E,RAET1E-AS1,RAET1G,ULBP2,ULBP1,RAET1K,RAET1L,ULBP3,PPP1R14C,IYD,PLEKHG1,MTHFD1L | - | - |
| chr8:39,359,817-39,533,168 | Loss | 10 | ADAM5,ADAM3A | - | - |
| chr8:0-1,452,246 | Loss | 18 | OR4F21,RPL23AP53,ZNF596,FAM87A,FBXO25,TDRP,ERICH1,LOC401442,ERICH1-AS1,LOC286083,DLGAP2 | - | - |
| chr9:8,381,903-10,135,588 | Loss | 11 | PTPRD-AS1,PTPRD | - | - |
| chr10:131,710,606-134,068,182 | Loss | 9 | LINC00959,CTAGE7P,GLRX3,MIR378C,TCERG1L-AS1,TCERG1L,LINC01164,PPP2R2D,BNIP3,JAKMIP3,DPYSL4,STK32C,LRRC27,PWWP2B | - | - |
| chr11:132,714,177-134,452,384 | Loss | 14 | OPCML,LOC646522,SPATA19,MIR4697,MIR4697HG,IGSF9B,LOC100128239,JAM3,NCAPD3,VPS26B,THYN1,ACAD8,GLB1L3,GLB1L2,B3GAT1,LOC283177 | - | - |
| chr13:110,713,667-111,877,154 | Loss | 7 | ARHGEF7,TEX29,LINC00354,LINC00403,SOX1 | - | - |
| chr14:19,323,579-19,564,886 | Loss | 8 | OR4N2,OR4K2,OR4K5,OR4K1,OR4K15,OR4K14 | - | - |
| chr15:18,362,555-19,906,749 | Loss | 13 | CHEK2P2,HERC2P3,GOLGA6L6,GOLGA8CP,NBEAP1,LOC646214,CXADRP2,MIR3118-2,MIR3118-3,MIR3118-4,POTEB2,POTEB,POTEB3,NF1P2,MIR5701-1,MIR5701-2,MIR5701-3,LINC01193,LOC727924,LOC101927079,OR4M2,OR4N4 | - | - |
| chr16:75,113,428-76,314,342 | Loss | 13 | CNTNAP4,LOC101928203,MIR4719,MON1B,SYCE1L,ADAMTS18,NUDT7 | - | - |
| chr16:33,454,467-34,903,000 | Loss | 8 | ENPP7P13,LINC00273,UBE2MP1,LINC01566,FRG2DP,TP53TG3HP,FLJ26245 | - | - |
| chr17:10,832,111-11,804,121 | Loss | 17 | SHISA6,DNAH9 | - | - |
| chr17:28,641,855-30,271,750 | Loss | 12 | AA06,ASIC2,LOC101927239,CCL2,CCL7,CCL11,CCL8,CCL13,CCL1,C17orf102,TMEM132E | - | - |
| chr18:5,961,056-7,125,051 | Loss | 10 | L3MBTL4,L3MBTL4-AS1,MIR4317,LINC01387,ARHGAP28,LINC00668,LOC101927188,LAMA1 | - | - |
| chr18:73,158,585-74,939,833 | Loss | 11 | LINC01029,SALL3,ATP9B | - | - |
| chr22:46,795,328-48,007,549 | Loss | 15 | MIR3201,LOC284933,FAM19A5,MIR4535,LINC01310 | - | - |
| chrX:5,127,672-8,738,437 | Loss | 11 | NLGN4X,MIR4770,VCX3A,PUDP,MIR4767,STS,VCX,PNPLA4,MIR651,VCX2,VCX3B,ANOS1,FAM9A | - | - |
| chrX:62,893,204-64,474,249 | Loss | 10 | ARHGEF9,MIR1468,AMER1,ASB12,MTMR8,ZC4H2 | - | - |
